# Supplementary material for: Nutritional Status and Physical Exercise Are Associated with Cognitive Function in Chinese Community-Dwelling Older Adults: The Role of Happiness
Source: Nutrients. 2024 Jan 8;16(2):203. doi: 10.3390/nu16020203 (PMC10819784; doi:10.3390/nu16020203)
Supplement: Supplementary file 1 [file nutrients-16-00203-s001.zip › nutrients-2775706-supplementary.pdf]

Supplementary Information

Nutritional Status and Physical Exercise Are Associated with Cognitive Function in Chinese Community-Dwelling Older Adults: The Role of Happiness

Jianghong Liu [1,*], Michael Pan [1,2], McKenna Sun [1,3], Haoer Shi [1,2] and Rui Feng [4]

[1] School of Nursing, University of Pennsylvania, Philadelphia, PA 19104, USA; pdyd@seas.upenn.edu (M.P.); masun@sas.upenn.edu (M.S.); haoershi@seas.upenn.edu (H.S.)
[2] School of Engineering and Applied Science, University of Pennsylvania, Philadelphia, PA 19104, USA
[3] College of Arts & Sciences, University of Pennsylvania, Philadelphia, PA 19104, USA
[4] Department of Biostatistics, Epidemiology, and Informatics, Perelman School of Medicine, University of Pennsylvania, Philadelphia, PA 19104, USA; ruifeng@pennmedicine.upenn.edu
* Correspondence: jhliu@nursing.upenn.edu; Tel.: +1-(215)-898-8293

Table S1. Bivariate Pearson's correlation between variables.

Predictors	Nutrition	Exercise	Happiness	Cognitive decline
Nutrition				
Exercise	0.15***			
Happiness	0.21***	0.15***		
Cognitive decline	-0.25***	-0.13***	-0.17***	
Age	-0.13***	-0.08*	-0.01	0.10

Note: Nutrition, MNA nutritional status; Exercise, physical activity, 1 = not doing exercise, 2 = doing exercise. *: p-value < 0.05, ***: p-value < 0.001.

Table S2. Adjusted association of cognitive decline with nutritional status, stratified by age.

Predictors	Young-old (N = 524) β (SE)	Old-old (N = 173) β (SE)
Nutrition (Normal as reference)		
At-risk	2.96 (0.95)**	2.90 (1.53)†
Malnutrition	11.10 (2.30)***	9.05 (5.61)
Exercise (No as reference)		
Yes	-2.35 (0.96)*	-1.16 (1.64)

Note: All models adjusted for age, gender, income, education, marital status. β, estimated regression coefficient; SE, standard error; *: p-value < 0.05, **: p-value < 0.01, ***: p-value < 0.001. † Indicated marginal significance.

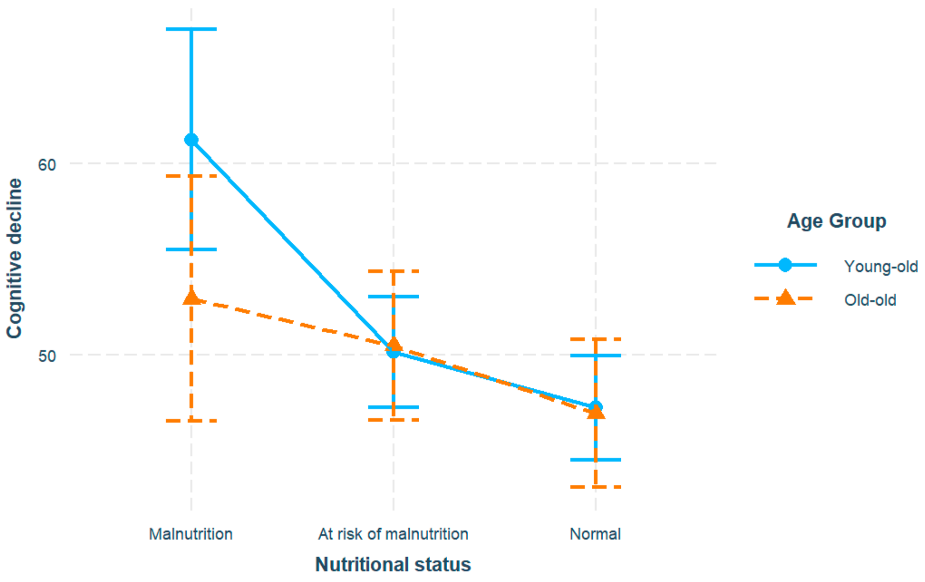

Figure S1. Stratified associations of cognitive decline with nutritional status across young-old (age < 74) and old-old (age ≥ 74) age sub-groups. Models adjusted for age, gender, income, education, marital status.
